# Development and Characterization of *n*-Propyl Gallate Encapsulated Solid Lipid Nanoparticles-Loaded Hydrogel for Intranasal Delivery

**DOI:** 10.3390/ph14070696

**Published:** 2021-07-19

**Authors:** Fakhara Sabir, Gábor Katona, Ruba Ismail, Bence Sipos, Rita Ambrus, Ildikó Csóka

**Affiliations:** 1 Institute of Pharmaceutical Technology and Regulatory Affairs, Faculty of Pharmacy, University of Szeged, Eötvös Str. 6, H-6720 Szeged, Hungary; fakhra.sabir@gmail.com (F.S.); katona.gabor@szte.hu (G.K.); ruba.ismail@szte.hu (R.I.); sipos.bence@szte.hu (B.S.); ambrus.rita@szte.hu (R.A.); 2 Department of Applied & Environmental Chemistry, Faculty of Science and Informatics, University of Szeged, Rerrich Béla sqr. 1, H-6720 Szeged, Hungary

**Keywords:** hydrogel, SLNs, nose-to-brain delivery, mucoadhesion, quality by design, antioxidant activity

## Abstract

The objective of the present study was to develop *n*-propyl gallate-loaded solid lipid nanoparticles (PG-SLNs) in a hydrogel (HG) formulation using Transcutol-P (TC-P) as a permeation enhancer. Modified solvent injection technique was applied to produce optimized PG-SLNs via the Quality by Design approach and central composite design. The in vitro mucoadhesion, scavenging activity, drug release, permeation studies of PG from PG-SLNs-loaded HG were evaluated under simulated nasal conditions. Compared with in vitro release behavior of PG from SLNs, the drug release from the PG-SLNs-loaded HG showed a lower burst effect and sustained release profile. The cumulative permeation of PG from PG-SLNs-loaded HG with TC-P was 600 μg/cm^2^ within 60 min, which is 3–60-fold higher than PG-SLNs and native PG, respectively. Raman mapping showed that the distribution of PG-SLNs was more concentrated in HG having lower concentrations of hyaluronic acid. The scavenging assay demonstrated increased antioxidant activity at higher concentrations of HG. Due to enhanced stability and mucoadhesive properties, the developed HG-based SLNs can improve nasal absorption by increasing residence time on nasal mucosa. This study provides in vitro proof of the potential of combining the advantages of SLNs and HG for the intranasal delivery of antioxidants.

## 1. Introduction

In the past few years, the intranasal administration route has gained considerable interest since it provides a non-invasive method to bypass the blood–brain barrier (BBB). Most regions in the central nervous system (CNS) can be directly reached along the olfactory and trigeminal nerves by intranasal administration of drugs. This intranasal administration route is broadly innervated by the olfactory nerve, which is localized in the epithelial tissue of the nasal olfactory mucosa and respiratory mucosa [1]. Various studies provide promising data for the potential of nose-to-brain delivery pathway in the treatment of CNS diseases such as brain tumors, Parkinson’s disease and Alzheimer’s disease [2,3].

Nose-to-brain delivery is considered very effective for many CNS active drugs that have limited administration because of low bioavailability through other delivery routes including paclitaxel, levetiracetam, cephalexin, dopamine, estrogen or even nerve growth factor-1 [4]. Limited brain uptake can be achieved in numerous intranasally applied compounds from conventional formulations, including chemotherapeutics and antineoplastic agents, due to their low permeability, enzymatic degradation and rapid elimination by mucociliary clearance from the nasal cavity [5,6,7]. To hurdle these obstacles, the application of nano drug delivery systems can be a suitable tool.

Lipid nanoparticles, including liposomes, niosomes, nanoemulsions and solid lipid nanoparticles (SLNs), are among the most promising drug delivery systems because of their biocompatible nature [8,9,10]. Moreover, these nanosystems can provide protection of the embedded active pharmaceutical ingredient (API) against efflux transporters (P-glycoprotein), enzymatic degradation or chemical destabilization at nasal conditions [11]. Compared with conventional lipid nanoparticulate drug delivery systems, active targeting has attracted significant attention due to enhanced therapeutic benefits and reduced undesirable side effects.

Hyaluronic acid (HA), a glycosaminoglycan, is a linear polysaccharide that is composed of β-1,4-d-glucoronic acid and 1,3-*N*-acetyl-d-glucosamine disaccharides via alternating glycosidic bonds [12,13,14]. Hydrogels (HG) containing HA have been successfully developed and evaluated for several promising biomedical applications as carrier systems in nasal, pulmonary, parenteral, topical and ophthalmic delivery [15,16]. Loading nanoparticles into low molecular weight HA-HG can improve nasal absorption by increasing residence time on nasal mucosa through enhanced viscosity and mucoadhesive properties. Improved mechanical stability against degradation and enhanced biochemical functionality of HA can be easily reached using cross-linkers such as glutaraldehyde (GA), divinyl sulfone, carbodiimide or bisepoxide [17,18]. In addition to the gel-forming properties, HA can also be applied in targeted drug delivery [19]. In our experiments, GA was applied due to having high potency to function as a proper cross-linker and because the nasal lining is fairly resistant to aldehyde toxicity below millimolar concentrations [20].

Propyl gallate (PG) (propyl 3,4,5-tri-hydroxybenzoate) is an ester form of gallic acid, and propanol functions as a synthetic antioxidant. Previous studies showed that PG has a high antioxidant capacity, which may contribute to decreasing mitochondrial impairment and to inhibiting cellular respiration. PG has demonstrated anticancer effects on various normal and tumor cells that may lead to DNA genotoxicity, cytotoxicity and fragmentation [21,22]. It has been revealed that PG used along with other anti-tumor agents such as probiotics was effective in mice for tumor treatment [23]. The PG anticancer activity can stop cell proliferation, reduce reactive oxygen species production and stimulate the autophagy of malignant cells [24].

This study aimed to optimize PG containing solid lipid nanoparticles (PG-SLNs) embedded into chemically linked HA-HG as a suitable delivery system for the intranasal route. Intranasal administration of PG can be a promising approach for targeted treatment of brain tumors, e.g., glioblastoma multiforme [25] bypassing the BBB; moreover, this non-invasive way of administration can be more favorable for patients. A further aim was to investigate the effect of excipients as a permeation enhancer for Transcutol-P (TC-P) due to its nontoxicity and biocompatibility and GA as cross-linker for intranasal route. For the formulation optimization, the Quality by Design (QbD) methodology was applied as a quality improvement principle that is able to take into account all critical parameters that have an impact on final product quality, safety and efficacy using a response surface quadratic model.

## 2. Results

### 2.1. Quality by Design Approach and Risk Assessment (RA)

Screening of the quality target product profile (QTPP) was based on previous experimental data and according to the relevant International Conference on Harmonization (ICH) guidelines (Q8,Q9,Q10,Q11) [26,27]. The QTPP elements in this study were the route of administration, indication, dissolution and permeability profiles, stability and brain distribution [28,29]. QTPPs contain the information required by the mentioned ICH guidelines on the one hand, and the basic assumptions that our product must meet on the other. Based on the QTPPs, the defined aim was to develop a monodisperse PG-containing SLN embedded in a HG formulation that is able to enter the central nervous system via the nose-to-brain pathway as a patient adherence improving drug delivery pathway offering direct transport to the CNS. Figure S1 shows the relations established between the QTPP-CQA (QTPP-critical quality attribute) and the critical process parameters/critical materials attributes (CPPs/CMA-CQA) elements on a 3-grade scale. During the RA process, the particle characteristics of the nanoformulation were placed under thorough evaluation as they are the key elements during the incorporation to an HG formulation.

**Figure 1:**
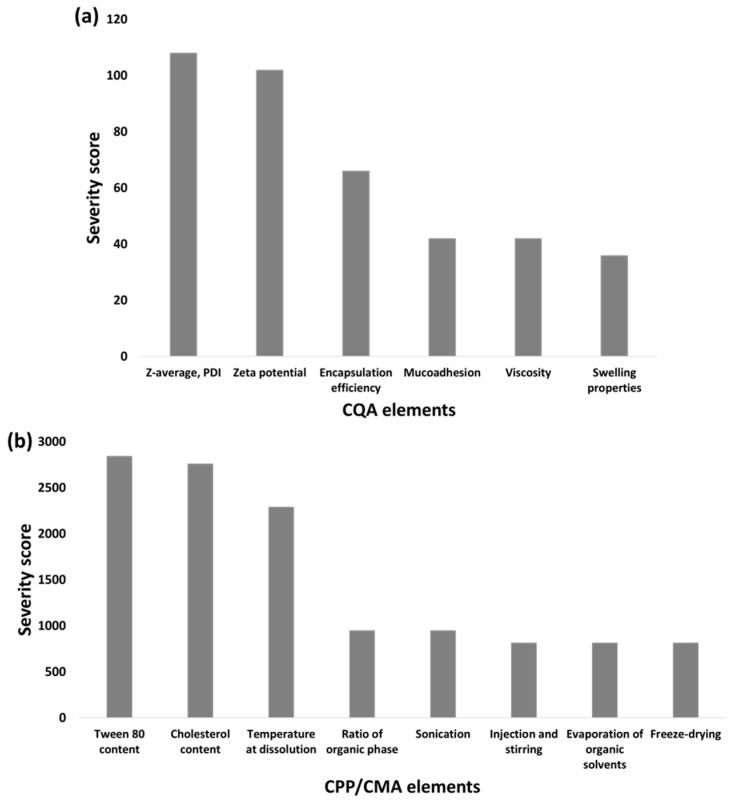
Probability rating of CQA (**a**) and CPP/CMA (**b**) elements. The Pareto charts are presented as the calculated severity scores assigned to the elements.

Based on the interdependence rating and using the software, quantification of these relations was performed, and severity scores were assigned for each CQA and CPP/CMA element, as presented in Figure 1.

The interdependence rating (Figure S1) assigned mostly high-grade scores concerning the relations of particle characteristics (Z-average, PDI and zeta potential) which is supported by the higher severity scores in Figure 1a compared with the applicability affecting risk factors such as muco-adhesivity, viscosity and swelling properties. The key element in QbD-driven nanoparticle formulation is to establish the basis for proper particle size and distribution, as these are the main elements influencing the dissolution and permeability profile, which are the first crucial steps in the nasal administration in the nasal cavity and through the nasal mucosa. Based on the calculations, it can be claimed that material attributes such as the concentration of Tween 80 and cholesterol hold the highest risk severity, followed by the temperature at dissolution phase compared with the subprocesses, as seen on Figure 1b. The subprocesses might hold low severity due to the fact that these are either easily controllable processes or because their main function is to achieve the final dosage form, whilst without the appropriate ratios and proportions of the material attributes, the nanosystem cannot be formed.

### 2.2. Central Composite Design (CCD)

#### 2.2.1. Optimization and Impact of Critical Parameters on Z-Average, Polydispersity Index (PDI), Zeta Potential

Based on the QbD methodology-based RA process, the design of the experiment was conducted according to severity scores. The effect of the characteristics with the highest severity score, i.e., the cholesterol content (A), the Tween 80 content (B) and temperature (°C), were investigated on the independent factors: Z-average, PDI and zeta potential in a 15-formulation experiment series presented in Table 1. We incorporated the results into the software, and as a result, a design expert selected a run (7) depending upon the smallest size, PDI and the characteristic with more negative zeta potential. The software screened the optimized trial with the desirability of 0.99 depending on the lowest Z-average, PDI and more negative zeta potential. The optimized PG-SLNs consisted of 1:6 of Tween 80 and cholesterol. The effects of each individual factor and the combined effect of factors on studied factors are shown in Figure S2a. It was revealed that at a low amount of cholesterol (20 mg), the Z-average was slightly higher than set trials where the maximum amount of it (60 mg) was used. With surfactant addition, the Z-average was less at low concentration of cholesterol due to lower lipid aggregation because of Tween 80 incorporation.

Even though smaller particle size was obtained at a higher temperature, the temperature effect was not statistically significant. The interaction of individual factors, i.e., cholesterol, on particle size, PDI and zeta potential, are also presented in Figure S2b.

The significance of the applied model was evaluated by *F*-value and *p*-value. In the case of particle size analysis, the model *F*-value of 56.89 implies that the model is significant. The following equations describe the linear and quadratic relations of the individual parameters influencing the dependent factors:

(1) $$\mathrm{Particle}\;\mathrm{size}=160.57-23.74A+144.53B-60.62C+16.88\mathrm{AB}-17.97\mathrm{AC}-50.11\mathrm{BC}+26.74A^{2}+176.21B^{2}-16.67C^{2}$$

In the case of zeta potential, the model *F*-value of 10.31 implies that the model is significant. There is only a 0.97% chance that a “model *F*-value” this large could occur due to noise. Values of “Prob > *F*” less than 0.0500 indicate that model terms are significant.

(2) $$\mathrm{Zeta}\;\mathrm{Potential}=-27.42-0.8A+11.08B+3.45C+8.7\mathrm{AB}-0.67\mathrm{AC}+2.8\mathrm{BC}+4.78A^{2}+8.2B^{2}-0.73C^{2}$$

In the case of PDI, the model *F*-value of 9.41 implies that the model is significant.

(3) $$\mathrm{PDI}=0.24-0.01A+0.0988B+0.028C+0.042\mathrm{AB}-0.057\mathrm{AC}+0.026\mathrm{BC}-0.012A^{2}+0.075B^{2}+0.019C^{2}$$

Regarding the particle size, B, C, B^2^ are significant model terms, while in the case of the Zeta potential and PDI, B, B^2^ are significant models. In all three cases, the values were less than 0.1000, indicating the significance of the applied model. The optimized PG-SLNs were further evaluated for encapsulation efficiency (EE), percentage yield and loading capacity (LC). Optimized PG-SLNs resulted in 84 ± 0.47% EE and 60 ± 0.03% LC. The yield of the PG-SLNs was up to 80 ± 0.1%. Z-average, PDI and zeta potential were also measured for optimized PG-SLNs. The Z-average of PG-SLNs was reported as 103 ± 46 nm with PDI of 0.16 ± 0.001 and zeta potential of −36 ± 4.78 mV.

#### 2.2.2. XRPD and FTIR Analysis

**Figure 2:**
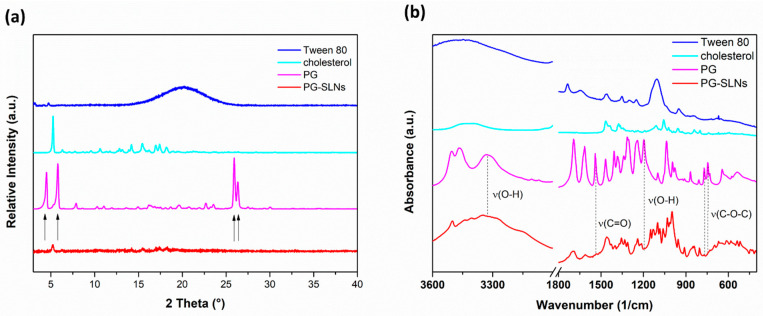
XRPD diffractogram (**a**) and FTIR spectra (**b**) of PG-SLNs and their components.

X-ray powder diffractograms (XRPD) of PG show sharp, characteristic peaks confirming its crystalline nature (Figure 2a). The results show that the characteristic peaks of PG (4.1°, 6.2°, 25.8° and 26.4° 2θ) could not be observed in the diffractogram of PG-SLNs, which shows that the crystalline structure of PG is converted into amorphous form and also supports the EE measurement data according to the high amount of PG that was successfully encapsulated into SLNs. In the diffractogram of PG-SLNs, only one peak can be detected at 5.2° 2θ, which corresponds to cholesterol as a carrier base material. These results of PG-SLNs are in agreement with similar experiments reported in the literature [30]. The FTIR spectra (Fourier-transformed infrared spectroscopy) of the components and the formulation are presented in Figure 2b. The characteristic peaks of PG including O–H stretching vibration at 3331 cm^−1^, C=O stretching of ester at 1539 cm^−1^, phenol O–H bending at 1246 cm^−1^ as well as C–O–C stretching of aromatic ester at 770 cm^−1^ and 745 cm^−1^ become unobvious in the spectrum of PG-SLNs, which also indicates the encapsulation of PG into SLNs. No other remarkable shift was observed in the other spectral regions, which supports that there is no interaction between SLNs and components.

### 2.3. Characterization of Hydrogels

#### 2.3.1. Evaluation of pH and Drug Contents of Hydrogels

It has been reported in several previous studies that lysozyme as a physiological nasal mucosa enzyme could inhibit specific types of microbes under slightly acidic conditions [31]. Therefore, the pH of an ideal nasal formulation should be in the range of 5.0 to 6.0 to preserve the physiological microbiological defense [32,33]. The pH of the HA-HGs was between 5.2 and 5.9, which is suitable for nasal administration. Drug content of HA-HGs was 78–82% *w*/*v* measured by HPLC, as shown in Table 2.

#### 2.3.2. Raman Chemical Mapping

**Figure 3:**
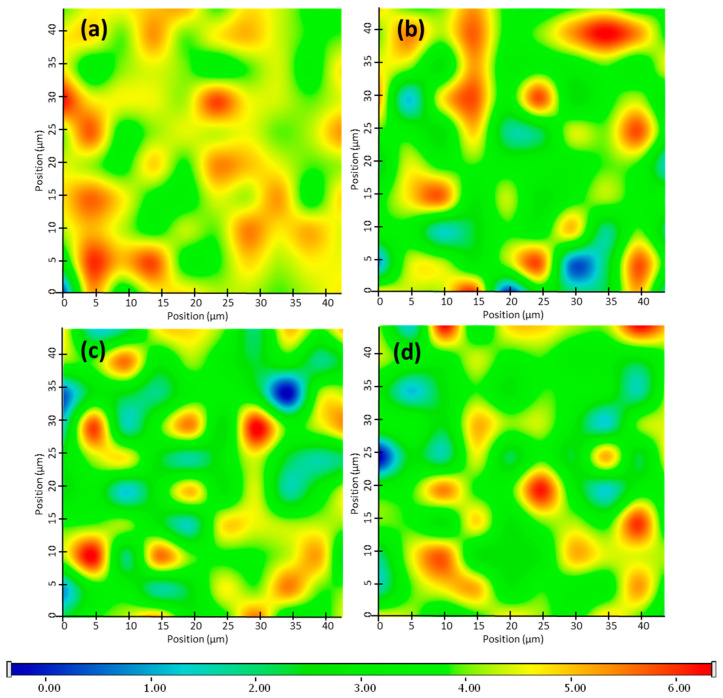
Raman chemical mapping of PG-SLNs in HGs with different concentration of HA: 0.5 (**a**), 1 (**b**), 2 (**c**) and 3% *w*/*v* (**d**).

Raman mapping was carried out in order to examine the distribution of PG-SLNs in non-cross-linked HA-HGs (SLNs-HGnCL) of different concentrations. For localization of nanoparticles, the Raman spectrum of PG-SLNs were set as profile, whose frequency of occurrence is shown in the chemical maps (Figure 3). The different colors of the chemical map show the relative intensity change of PG-SLNs in the gel structure. Red color indicates strong existence of PG-SLNs, whereas blue color marks those regions of the map whose spectral resolution contains different spectra, characteristic for another component. The results reveal that the distribution of PG-SLNs is more concentrated in HGs containing HA in lower concentration (0.5% and 1% *w*/*v*), as shown by high relative intensity values of the Raman map, whereas in the case of higher HA concentration (2% and 3% *w*/*v*), PG-SLNs can be found in well-defined packages.

#### 2.3.3. Spreadability and Swelling Studies of Hydrogel

**Figure 4:**
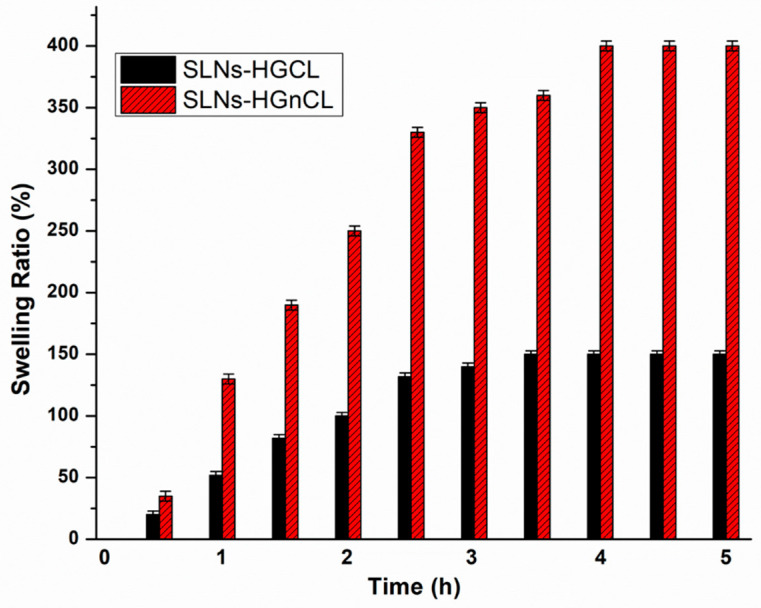
Swelling study of optimized cross-linked (SLNs-HGCL) and non-cross-linked (SLNs-HGnCL) HG (1% *w*/*v* HA). Data are means ± SD (*n* = 3 independent measurements).

The spreadability values of both cross-linked (SLNs-HGCL) and non-cross linked (SLNs-HGnCL) SLNs-HGs was investigated. No significant effect of cross-linking was observed in case of spreadability; SLNs-HGs with different concentrations of HA (0.5, 1, 2 and 3% *w*/*v*) showed 222.45 ± 0.22, 360.10 ± 0.33, 320.12 ± 0.44 and 340 ± 0.01 mm^2^ spreading surface, respectively (Table 2). Values in this range ensure proper spreading of the hydrogel. The spreadability study showed that the optimized SLNs-HG (1% *w*/*v* HA) resulted in the highest spreading surface [34,35]. Swelling studies were performed both with cross-linked and non-cross-linked hydrogels. The results show that all the non-cross-linked formulations show a higher swelling index than the chemically cross-linked HG, which can be claimed with the hindered diffusion of water into the cross-linked HG network (Figure 4). The formulation’s property to spread uniformly and easily on an applied surface is important to deliver a uniform dose of the active compound.

#### 2.3.4. Viscosity Measurement

**Figure 5:**
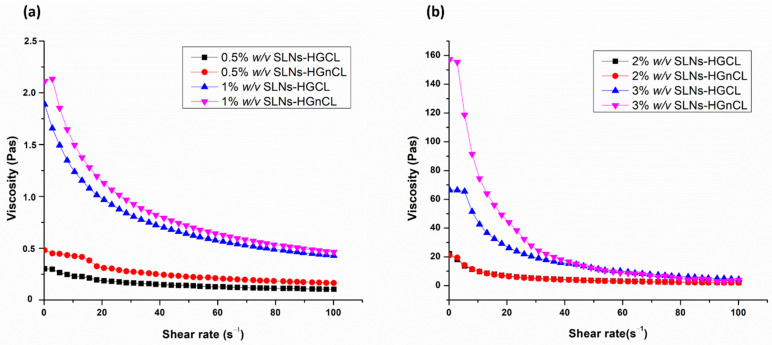
Viscosity profiles of cross-linked (SLNs-HGCL) and non-cross-linked (SLNs-HGnCL) containing HA in 0.5–1% *w*/*v* (**a**) and 2–3% *w*/*v* (**b**) concentration. Data are means ± SD (*n* = 3 independent measurements).

Viscosity is of paramount importance in the case of the applicability of hydrogels to the intranasal administration route, which influences the mucoadhesive properties of the formulation and can prolong the residence in the nasal cavity. Proper polymer concentration must be set in order to achieve the desired high viscosity value, allowing increased residence time and making it possible to enhance the absorption through the nasal mucosa. However, too high viscosity can also be disadvantageous, resulting in hindered drug release of the formulation in the nasal cavity. The viscosity of both cross-linked and non-cross-linked HGs was measured (Table 2). The viscosity measurement of all formulations showed that there was an inverse relation between shear rate and viscosity of HG, which proves the thixotropic nature of HGs (Figure 5). No significant differences among viscosities of cross-linked and non-cross-linked HGs was observed. Furthermore, the non-cross linked SLNs-HGs were characterized due to the spreadability investigation, where SLNs-HGnCL formulations showed higher swelling ratio and spreadability, along with the non-significant difference experienced in the viscosity compared with cross-linked SLNs-HGs.

#### 2.3.5. In Vitro Mucoadhesion Study

**Figure 6:**
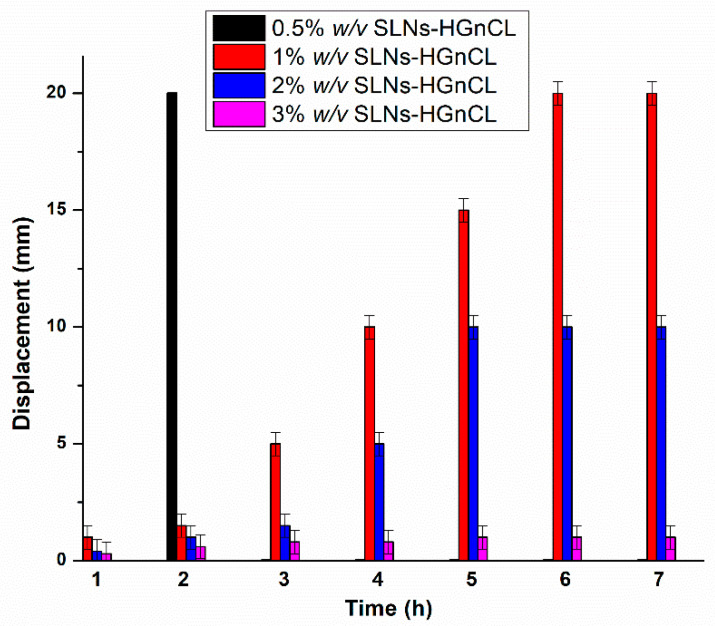
In vitro mucoadhesive studies of SLNs-HGs on agar-mucin gel. Data are means ± SD (*n* = 5 independent measurements).

The in vitro mucoadhesion of HGs was investigated through their displacement on an agar-mucin plate. Figure 6 shows that the adhesion potential of HGs at different concentrations of HA is inversely related to the displacement of the HG. As the polymer (HA) concentration increased, lower displacement was observed on the surface of agar after 7 h, indicating higher mucoadhesive properties of formulation. This can be related to the increasing strength of chemical interactions (secondary bonding) between mucin and HA. At the highest concentration (3% *w*/*v*) of HA, hardly any displacement was observed during the studied time, indicating remarkable mucoadhesion of formulation. At a lower concentration (0.5% *w*/*v*) of HA, displacement was not adequate for nasal administration, and after 2 h it was totally displaced from the agar-mucin plate. Based on these results, the optimized concentration of HG (1% *w*/*v*) was screened out, as displacement measured at this concentration was adequate and also in accordance with the results of viscosity, spreadability and swelling ratio measured at this specific concentration, as shown in Table 2.

#### 2.3.6. Morphological Study of PG-SLNs and PG-SLNs-Loaded HG

**Figure 7:**
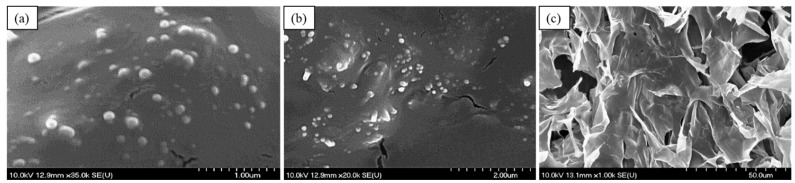
SEM images of optimized lyophilized PG-SLNs (**a**,**b**) and 1% *w*/*v* SLNs-HGnCL (**c**) at various resolution.

SEM images of lyophilized PG-SLNs, shown in Figure 7, also proves that the nanoparticles have spherical morphology and are homogenously distributed in the gel structure. The SEM image of optimized freeze-dried 1% *w*/*v* SLNs-HGnCL shows porous structure with a dense cross-linking network.

#### 2.3.7. In Vitro Permeation

**Figure 8:**
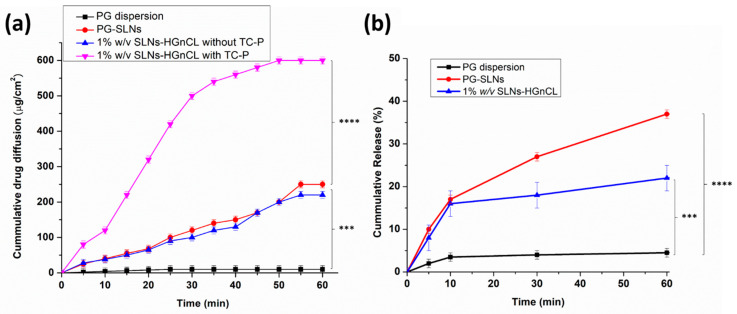
In vitro permeation of pure PG dispersion, PG-SLNs, 1% *w*/*v* SLNs-HGnCL with and without TC-P (**a**). In vitro release study of PG dispersion, PG-SLNs, 1% *w*/*v* SLNs-HGnCL (**b**). Data are means ± SD (*n* = 3 independent measurements). *** *p* < 0.001, **** *p* < 0.0001.

Modified Side-Bi-Side^®^ apparatus was used for the in vitro nasal permeation study, whereas the diffusion of PG solution, PG-SLNs, as well as TC-P containing and TC-P-free 1% *w*/*v* SLNs-HGnCL was compared. The TC-P was used as permeation enhancer due to its nontoxic and biocompatible nature. Several studies reported its permeation-enhancing effect through a synthetic membrane and on excised skin, reported in previous studies with different drugs [31,36,37,38]. Figure 8a shows the cumulative PG permeation from donor to acceptor phase through a synthetic cellulose membrane impregnated with isopropyl myristate. The cumulative permeation of PG from PG-SLNs was ~190 µg/cm^2^ after 60 min. The TC-P-free 1% *w*/*v* SLNs-HGnCL showed a lower permeation of about 180 µg/cm^2^, which supports the advantage of application of permeation enhancer. In the case of pure PG dispersion, the permeation was lower than 10 µg/cm^2^. The significantly highest permeability was achieved with the HG containing TC-P reaching, with a value around 600 µg/cm^2^.

#### 2.3.8. In Vitro Release Study

In vitro release studies of PG-SLNs showed burst release of drug in the first 60 min (~40% of drug) at pH 5.6. After that, the drug release rate decreased following a sustained release tendency, as shown in Figure 8b. In the case of the HG formulation, the initial burst effect was lower, which can be claimed with the controlled release effect of the gel matrix. After 60 min only 15% of PG was released from the HG. To determine the release kinetic of PG from SLNs as well as HG, various dissolution kinetic models including zero-order, first-order, Higuchi, Korsmeyer–Peppas and Hixon–Crowel were fitted to the release data, and kinetic parameters were calculated (Table S1). The drug release both from SLNs and HG followed Higuchi kinetics (*R*^2^ = 0.96 and 0.9783 respectively), which can be claimed with the drug release controlling mechanism of lipid matrix and swelling ability of HA-PG matrix in the simulated nasal medium [39]. Fitting the Korsmeyer-Peppas model, the “*n*” value was lower than 0.5, which indicates that both formulations follow Fickian drug diffusion. The significant difference between SLNs and HG can be claimed with the presence of gel network surrounding the SLNs, which decreases the velocity of Fickian diffusion, resulting in sustained release of PG.

#### 2.3.9. In Vitro Antioxidant Activity Evaluation with Hydrogen Peroxide Scavenging Assay

**Figure 9:**
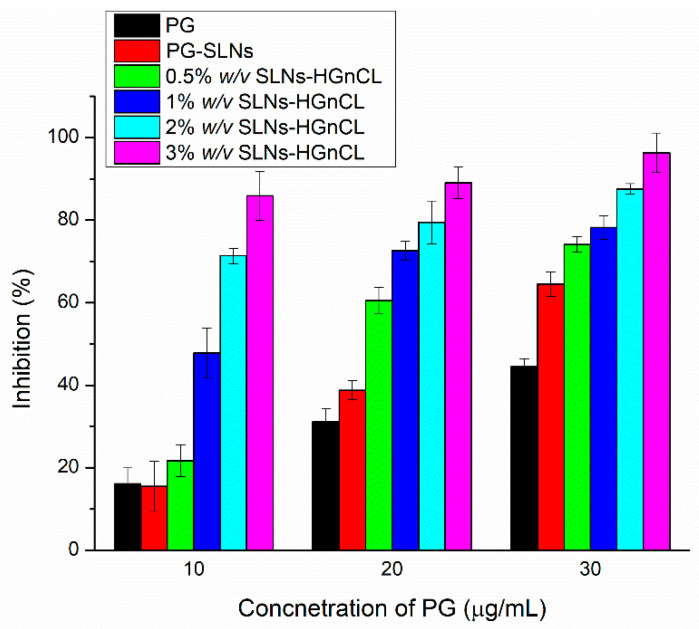
Percentage inhibition of the hydrogen-peroxide-scavenging activity of different concentrations of PG-containing SLNs and PG-SLNs-loaded HA-HG in comparison with the initial PG solution. Data are means ± SD (*n* = 3 independent measurements). Statistical analysis: *t*-Test.

The antioxidant activity of PG was investigated, aiming to confirm that PG-SLNs embedded in HA-HG can preserve antioxidant activity, which is essential in their pharmacological effect. It has been demonstrated that free radicals have a pivotal role in the pathogenesis of different diseases, as well as in several types of cancer [40]. The in vitro scavenging activity of PG-SLN-loaded HA-HGs was investigated containing PG in different concentrations utilizing hydrogen peroxide (H_2_O_2_) as oxidative medium (Figure 9). It has been revealed that by increasing the polymer concentration in the HG, the antioxidant activity of PG against H_2_O_2_ was improved, which can be explained by the protective effect of the hydrogel network. The results also support that a strong correlation could be found between the concentration of PG and the rate of inhibition of scavenging activity of H_2_O_2_. By increasing the concentration of PG, the inhibition was enhanced. At 10 and 30 μg/mL PG containing HGs (containing 2% and 3% *w*/*v* HA), the antioxidant activity was significantly higher in comparison with low HA concentration HGs and PG-SLNs or PG control, which also supports the stabilizing effect of the polymer matrix. In the case of 20 μg/mL, the same tendency of difference was also demonstrated, but it was not significant.

## 3. Discussion

In our previous work we had already investigated the formulation possibilities of PG-loaded liposomes coated with HA [41], as PG has advantageous effects (anti-inflammatory, antioxidant and anticancer activity) in the treatment of brain tumors, e.g., glioblastoma multiforme. As PG is a water-insoluble compound, loading it into a nanocarrier can enhance its water solubility and drug release profile in different administration routes. The present study focused on the formulation of PG-SLNs and loading into HA-HG for brain targeting through intranasal administration. The novelty of our work lies in the fact that our research team explored the first-time application of the PG-SLNs-loaded HA-HG for intranasal delivery route.

SLNs were developed in our study due to their advantageous properties (biocompatibility, increased solubility, protection of drug and permeability enhancement) and loaded into HA-HG as a secondary carrier for facilitating drug transport via the intranasal route. Due to its mucoadhesive property, the role of HA is vital for intranasal drug delivery systems because the nasal cavity is subjected to mucociliary clearance [42,43]. TC-P was utilized as a permeation enhancer as it was previously successfully used as a surfactant or co-surfactant for intranasal application. TC-P has adequate solubilizing property, and it has the ability to enhance the drug’s solubility by orders of magnitude compared with other penetration enhancers.

Aiming to develop novel PG-SLNs-loaded into the HA-HG system, the QbD approach was applied. After the determination of the QTPP elements, the relations between CQA and CMA/CPP elements were evaluated, and a risk order was set up based on the software-calculated severity scores. By optimizing the factors having the highest severity via central composite design, the optimized SLNs had a Z-average of 120 ± 8.8 nm, with a PDI of 0.12 ± 0.08 and a zeta potential that was a more negative −38 ± 10.2 mV. The indirect and direct co-effect of cholesterol and Tween 80 on Z-average can be clearly seen in both the single factor and 3D plot (Figure S2a,b). The results show weak repulsion forces between lipid nanoparticles and the surfactant at lower concentration of cholesterol. However, at higher lipid concentrations, prominent repulsion forces can be observed, which can be explained by the presence of the surfactant phase between lipid nanoparticles and the decrease in van der Waals forces, all reducing the aggregation tendency of lipid droplets [44]. The surfactant concentration showed higher impact on the reduction of Z-average and PDI as compared with cholesterol. These results are in accordance with those reported by Azhar Shekoufeh Bahari et al. and Severino et al. [39,45]. The low Z-average and narrow PDI indicate improved nasal absorption, while the highly negative zeta potential due to surface properties of cholesterol supports high stability of formulation. The absolute value of zeta potential was higher than 30 mV, which ensures sufficient repulsive forces to attain better physical and colloidal stability of the nanosystem [46,47]. The morphological studies by SEM showed spherical shaped SLNs with homogenous distribution and nano-range particle size.

Raman mapping showed that the increasing concentration of polymer forms well-designed hydrogel matrix embedding PG-SLNs homogenously, indicating higher stability of nanoparticles avoiding aggregation [48,49,50]. The swelling study revealed that the polymeric chains were more flexible in cross-linker-free HG, ensuring water diffusion into the gel matrix.

In the case of chemically cross-linked HG, the free association of the polymer chain is hindered, resulting in a decreased swelling ratio. From these results, it can be concluded that by adding a cross-linker, the mechanical strength of the HG was enhanced. More precise controlled release of the nano-carrier from the gel matrix was reached as compared with simple HG, which is mechanically fragile; thus, larger pores may be created through which nano-carriers can easily liberate the active substance initiating a burst release. Previous studies already reported the similar effect of GA with chitosan HG [51,52,53]. Safety application of GA is based on previous studies, which showed the nose is very resistant to the aldehydes requiring the application of millimolar concentrations before toxic responses [30].

Our results indicate the significant potential of TC-P in enhancing the permeation of PG-SLNs across the artificial cellulose membrane from the HG matrix. TC-P has a greater influence on the thermodynamic driving force. The maximum thermodynamic driving force occurring at saturation is based on Fickian diffusion, according to which the concentration gradient is formed from a high concentration region to a region of lower concentration phase [36]. TC-P was previously used as a surfactant, co-surfactant and permeation enhancer for intranasal delivery in different micro- and nanoemulsions. Several studies demonstrated the influence of TC-P alone or in combination with propylene glycol on clonazepam permeation both in vitro and ex vivo via application of carbomer HGs. Mura et al. revealed that applying TC-P in the concentration range of 10–50% *w*/*w* increased skin penetration of the drug [42].

The anticancer activity of PG is based on its antioxidant property of removing free radicals [54]. The most important mechanism to achieve this goal is to donate hydrogen to free radicals and convert them into nonreactive species. PG can inhibit cellular damage mainly through their free radical scavenging property [40]. Therefore, we aimed to evaluate the antioxidant activity; the hydrogen peroxide (H_2_O_2_)-scavenging assay results showed enhanced antioxidant activity in case of applying PG as well as HA in higher concentrations. The developed PG-SLNs-loaded HA-HG with the TC-P system, by controlling the drug release, increasing the physical stability and enhancing the drug permeability via nasal mucosa while protecting its antioxidant activity, imparts a promising carrier system for the brain targeting of antioxidant drugs.

## 4. Materials and Methods

### 4.1. Materials

PG, the model antioxidant compound, HA (Mw = 14 kDa) and Tween 80 were purchased from Sigma-Aldrich (Budapest, Hungary). Cholesterol, acetone, ethanol (96% *v*/*v*), glutaraldehyde (25% *w*/*v*) and sodium chloride for physiological salt solution were purchased from Molar Chemicals Ltd. (Budapest, Hungary). Transcutol-P (diethyl glycol monoethyl ether) was supplied by Gattefossé (saint-Priest, France).

### 4.2. Optimization of SLNs by Quality by Design (QbD) Approach and Risk Assessment Strategy

First, the quality target product profile (QTPP) was defined, followed by selecting the critical quality attributes (CQAs) and critical process parameters (CPPs). The next step was to perform the risk assessment (RA) [55,56,57]. At first, an interdependence rating was established between the QTPP and CQA elements as well as between CPPs and CQAs. The RA was conducted using Lean QbD^®®^ software (QbDworks.com, QbD works LLc., Fremont. CA, USA). Each factor was thoroughly evaluated on a 3-grade scale using low (“L”), medium (“M”) and high (“H”) attributives reflecting the relations between the elements. Based on the interdependence rating, the next step was to quantify the severity of the risk factors via a probability rating. As a result of the RA, the severity scores of CQAs and CPPs were plotted on Pareto diagrams generated by the software.

### 4.3. Response Surface Quadratic Model

Stat-Ease Design Expert^®^ version 10 (stat-Ease, INC.2021 East Hennepin Ave., Suite 480 software) was used to optimize the formulation process and product quality of PG encapsulated SLNs. The amount of cholesterol (20–60 mg), Tween 80 (10–40 mg) and temperature (20–70 °C) where chosen as independent factors based on the RA process, while the ratio of aqueous to organic phases (acetone:ethanol) was kept constant (1:4). Central composite design was applied where SLNs were prepared for each trial, and three responses were evaluated—namely, average hydrodynamic diameter (Z-average), polydispersity index (PDI) and zeta potential.

### 4.4. Development of PG-SLNs by Modified Injection Method

PG-SLNs were prepared through the modified injection method, where 10 mL of 0.2% *w*/*v* Tween 80 aqueous solution and 5 mL of the organic phase (ethanol:acetone 4:1) were added in different ratios. The PG (10 mg) and cholesterol (60 mg) was dissolved in a mixture of the organic phase and injected dropwise into surfactant solution under constant stirring at 700 rpm at 70 °C. After complete evaporation of the organic phase, the formulation was purified using a Hermle Z323K high performance refrigerated centrifuge (Hermle AG, Gossheim, Germany) for 2 h at 13,500 rpm to separate pellets from supernatant and residual solvent. The collected pellets were redispersed in 5 mL purified water and freeze-dried using a Scanvac CoolSafe laboratory freeze-dryer (Labogene, Lynge, Denmark) at −40 °C for 12 h under a 0.013 mbar pressure with additional 3 h secondary drying at 25 °C in presence of 5% *w*/*v* trehalose as cryoprotectant to obtain lyophilized powders. The lyophilized powder was stored at 5 ± 3 °C until further investigation.

### 4.5. Preparation of Mucoadhesive HA-Based Hydrogel Formulations with PG-SLNs

Different concentrations (0.5, 1, 2 and 3 *w/v*) of HA were used to prepare colloidal HA-HGs, dissolving them in water for half an hour at 400 rpm. After complete swelling of the HA, the freeze-dried SLNs and 0.1% *w*/*v* glutaraldehyde as cross-linker were added into the HA to form an acetal bond among the aldehyde and hydroxyl group by maintaining acidic conditions. After the reaction, 1 mL of TC-P, as permeation enhancer, was added to the HGs.

### 4.6. Characterization of PG-SLNs

#### 4.6.1. X-ray Powder Diffraction (XRPD)

Structural characterization of PG-SLNs was performed using a BRUKER D8 Advance X-ray powder diffractometer (Bruker AXS GmbH, Karlsruhe, Germany). All the components and formulations were analyzed in a quartz sample holder and were scanned at 40 kV and 40 mA with Cu K λI radiation (λ = 1.5406 Å) using a VANTEC-1 slit detector in the angular range of 3° to 40° 2θ, at a step time of 0.1 s and an increment of 0.007°. Each measurement was carried out at ambient humidity and temperature.

#### 4.6.2. Fourier-Transformed Infrared Spectroscopy (FTIR)

The compatibility and interactions between PG and the components of the formulation were investigated using a Thermo Nicolet AVATAR FTIR instrument (Thermo-Fisher, Waltham, MA, USA). For the investigation, pellets were prepared by co-grinding 10 mg compound with 150 mg potassium bromide (KBr) and compressed with 10 tons using a hydraulic press. The FTIR spectra were measured over the range of 4000–400 cm^−1^ with a resolution of 4 cm^−1^ for 128 scans. The recorded spectra were reported as absorbance as a function of wavenumber.

#### 4.6.3. Measurement of Z-Average, Surface Charge and Polydispersity Index

The measured amount (6 mg) of lyophilized SLNs was reconstituted in 6 mL of purified water and sonicated for 4 min to minimize the inter-particle aggregation. The Z-average, PDI and surface charge (zeta potential) of PG-SLNs were measured in folded capillary cells using a Malvern Zetasizer nano ZS instrument (Malvern Instrument, Worcestershire, UK) at 25 °C, with the refractive index 1.445. All the measurements were conducted in triplicate, and data are presented as average ± SD.

#### 4.6.4. Encapsulation Efficiency (EE), Loading Capacity (LC) and Percentage Yield Determination

EE and LC of PG-SLNs were determined using an indirect method. PG-SLNs were first centrifuged for 1 h at 16,500 rpm at 4 °C in a Hermle laboratory centrifuge (Hermle AG, Gosheim, Germany). The supernatant was collected and diluted 10-fold with purified water. The concentration of PG was determined using HPLC. The EE, as the actual PG content in the optimized formulation, was measured according to the following equation:

(4) $$\mathrm{Encapsulation}\;\mathrm{Efficiency}\;\left(\%\right)=\frac{w_{1}-w_{2}}{w_{1}}\times 100$$

where w_1_ is the total amount of added PG, and w_2_ is the amount of free PG in the supernatant.

The LC was calculated via the following equation:

(5) $$\mathrm{Loading}\;\mathrm{capacity}\;\left(\%\right)=\frac{w_{1}-w_{2}}{w_{3}}\times 100$$

where w_1_ is the total amount of added PG, and w_2_ is the amount of free PG in the supernatant, while w_3_ is the total amount of lipid added to the formulation.

The percentage yield was calculated by weighing the dried PG-SLNs and determined by using the following formula [58]:

(6) $$\mathrm{Percentage}\;\mathrm{yield}\;\left(\%\right)=\frac{\mathrm{weight}\;\mathrm{of}\;\mathrm{dried}\;\mathrm{NPs}}{\mathrm{theoretical}\;\mathrm{weight}\;\mathrm{of}\;\mathrm{drug}\;\mathrm{and}\;\mathrm{components}}\times 100$$

#### 4.6.5. HPLC Method

The PG quantification was carried via HPLC (Agilent 1260, agent technologies, Santa Clara, CA, USA). The stationary phase was C18 column (Gemini-NX^®^ 150 mm × 4.6 mm, 5 µm (Phenomenex, Torrance, CA, USA). Purified water and acetonitrile in 80:20 ratio adjusted pH = 3.0, with phosphoric acid used as mobile phase. A set of 20 μL samples were injected, whereas separation was performed by 10 min isocratic elution at 25 °C temperature with 1 mL/min eluent flow. The UV-Vis diode array detector was applied for the detection of chromatograms at 254 nm. ChemStation B.04.03 Software (Santa Clara, CA, USA) was used for evaluation of data. PG retention time was detected at 4 min. For the calibration line, the linear regression was 0.998. The quantification limit (LOQ) and detection (LOD) of PG were 63 ppm and 21 ppm, respectively [41,59].

### 4.7. Characterization of PG-SLNs Loaded Hydrogels

#### 4.7.1. Physical Appearance, pH and Drug Contents of Hydrogels

PG-SLN HGs containing HA in different concentrations (0.5, 1, 2 and 3% *w*/*v*) were evaluated apparently for grittiness and uniformity. The pH of HGs was measured directly by dipping pH meter (WTW^®^ inoLab^®^ pH 7110 laboratory pH tester, Thermo Fisher Scientific, Budapest, Hungary). To determine the drug contents in the formulation, 1 g of HG was dispersed in 10 mL of phosphate buffer (pH 7.4). This diluted gel was filtered with a membrane filter (0.45 μm, polypropylene) and analyzed by HPLC. All measurements were carried out in triplicate. Drug content was evaluated by the following formula:

(7) $$\mathrm{Percent}\;\mathrm{Drug}\;\mathrm{contents}=\frac{\mathrm{Actual}\;\mathrm{amount}\;\mathrm{of}\;\mathrm{drug}\;\mathrm{in}\;\mathrm{the}\;\mathrm{formulation}}{\mathrm{Theoretical}\;\mathrm{amount}\;\mathrm{of}\;\mathrm{drug}\;\mathrm{in}\;\mathrm{thr}\;\mathrm{formualtion}}\times 100$$

#### 4.7.2. Raman Spectroscopy

A Thermo Fisher DXR Dispersive Raman instrument (Thermo Fisher Scientific Inc., Waltham, MA, USA) was used for investigation of SLNs. This instrument was equipped with a 780 nm wavelength diode laser and a CCD camera. The laser power of 12 mW at 50 µm slit aperture size was used for Raman measurements with 2 and 6 s of exposure and acquisition time, for a total of 32 scans per spectrum in the spectral range 3500–200 cm^−1^ with fluorescence and cosmic ray corrections. The PG-SLNs distribution in HGs was determined via Raman chemical mapping in the formulation. For the total of 16 scans, a 45 µm × 45 µm size surface was evaluated with a 10 µm step size. To eliminate the intensity deviation between the measured areas, the normalization of Raman spectra was ensured [60].

#### 4.7.3. Swelling Index

To measure the swelling index of the HGs, the gravimetric method was applied. Lyophilized gel of both cross-linked and non-cross-linked HGs was soaked in phosphate buffer saline (PBS) (pH = 7.4). After swelling, HGs were taken out from the medium and weighed at different time intervals (after every 30 min) until the weight of the swelled HG became constant. Percentage swelling was calculated using the formula [58]:

(8) $$\mathrm{Swelling}\;\left(\%\right)=\frac{w_{2}-w_{1}}{w_{1}}\times 100$$

where w_1_ is the initial weight of hydrogel, and w_2_ is the weight of swollen HG after each sampling point.

#### 4.7.4. Spreadability Test

Spreadability of HGs was measured using the glass slide method. The center of the glass slide was marked with a 1 cm diameter circle upon which 0.5 g of gel was placed. Another glass slide was placed over the HG, forming a sandwich arrangement. The load of the 500 g was placed on the upper plate and weighted for 5 min. After 5 min, the load was removed, and the increment in HG diameter was measured [61]. All the results were evaluated with respect to the spreading area and applied weight by using the following equation:

(9) $$s_{i}=d^{2}\times \frac{\pi \;}{4}$$

where S_i_ is the swelling index, d the diameter of the glass slide and π the shear stress.

#### 4.7.5. Viscosity Measurement

Viscosity measurement was performed at 37 °C with a Haake Rheostress 1 instrument (Thermo Scientific, Karlsruhe, Germany). A cone-plate device was used where the cone diameter was 6 cm with an angle of 1° and a 0.052 mm gap size. The apparent viscosity curves of the samples were plotted under the shear rate range of 0.01–100 s^−1^.

### 4.8. In Vitro Characterization of Nanoparticles and Hydrogel

#### 4.8.1. In Vitro Mucoadhesion Testing

In vitro mucoadhesion was performed using the displacement method. A specified weighted amount (5 mg) of HG for each respective HA concentration (0.5, 1, 2 and 3% *w*/*v*) was placed on the top of 1% *w*/*v* agar and 2% *w*/*v* mucin aqueous solution casted on a glass plate of 9 cm and was inclined at 60° in an incubator at 37 °C. The downward movement of the HG mass was measured in millimeters hourly up to 7 h. All the measurements were conducted in triplicate [61,62].

#### 4.8.2. Surface Morphology

Scanning electron microscopy (SEM) (Hitachi S4700, Hitachi Scientific Ltd., Tokyo, Japan) was used to characterize the morphology and surface properties of both PG-SLNs-lyophilized formulation and PG-SLNs-loaded HG. A voltage of 10 kV and 10 mA amperage was applied at 1.3–13.1 mPa pressure. A greater vacuum evaporator and argon atmosphere were used to make the sputter-coated samples conductive with gold–palladium (Bio-Rad SC 502, VG Microtech, Uckfield, UK). The gold–palladium coating thickness was approximately 10 nm.

#### 4.8.3. In Vitro Permeation Study

A modified horizontal side-by-side type diffusion apparatus was used for in vitro permeation studies at 37 °C and with 100 rpm constant stirring (Thermo Haake C10-P5, Sigma-Aldrich Co. LLC, St. Louis, MO, USA). The donor and receptor compartments were isolated with an isopropyl myristate impregnated artificial membrane (0.45 µm pore size, Pall Metri-cel cellulose membrane) with a 0.69 cm^2^ diffusion surface. The donor compartment consisted of 9 mL simulated nasal electrolyte solution (SNES) with a pH of 5.6, which contained 0.59 g CaCl_2_, 8.77 g NaCl, 2.98 g KCl anhydrous in 1000 mL of deionized water, where the acceptor compartment consisted of pH 7.4 PBS. The measured amount (5 mg) of formulation was placed in the donor phase 1 mL of each sample (PG, PG-SLNs, PG-SLNs-loaded HG with and without TC-P) and was withdrawn from the acceptor phase every 5 min and replaced with the same volume of fresh medium. The amount of the drug diffused through the membrane was quantified by using HPLC. Each formulation was analyzed in triplicate.

#### 4.8.4. In Vitro Release Study

The in vitro dissolution study was performed under the nasal conditions at 37 °C by using the modified paddle method with a Hanson SR8 Plus apparatus (Teledyne Hanson Research, Chatsworth, CA, USA) at 50 rpm constant stirring. The PG (10 mg) containing formulation was placed into 50 mL SNES (pH 5.60) as dissolution medium, and samples were withdrawn in predetermined time intervals of 5, 15, 30, 60, 180, 360 and 720 min. After filtration (0.45 μm membrane filter), the PG concentration of the aliquots was analyzed using HPLC. All the measurements were performed in triplicate. The in vitro drug release kinetics of each sample were also evaluated, fitting various mathematical models—namely, zero-order, first-order, Higuchi, Korsmeyer–Peppas and Hixon–Crowel models.

#### 4.8.5. Hydrogen Peroxide Scavenging (H_2_O_2_) Assay

A solution of hydrogen peroxide (40 mM) was prepared in 0.05 M phosphate buffer (pH 7.4). PG SLNs with different drug concentrations (10, 20 and 30 μg/mL) were incorporated in a 0.6 mL and 40 mM hydrogen peroxide solution. After 10 min of addition of hydrogen peroxide, the absorbance at wavelength of 230 nm was determined spectrophotometrically using phosphate buffer as reference [40,61,63]. The hydrogen peroxide percentage scavenging activity was then measured using the following formula:

(10) $${\;H}_{2}O_{2\;}\mathrm{scavenging}\;\mathrm{effect}\;\left(\%\right)=A_{0}-\frac{A}{A_{0}}\times 100$$

where *A_o_* is the absorbance of the control reaction, and *A* is the absorbance in the presence of initial PG containing sample.

### 4.9. Statistical Analysis

The statistical analysis was applied to all the results using Microsoft^®^ 13 software (SAS Institute, Cary, NC, USA). All the results were repeated in triplicate, and the means of data are expressed with standard deviation. In vitro permeation and release data were compared using the one-way analysis of variance (ANOVA); differences were considered significant when *p* < 0.05.

## 5. Conclusions

PG-SLNs were successfully prepared using a modified injection method. For the optimization of formulation and process parameters affecting the quality of the nanosystem, the initial risk assessment study and the design of experiment were applied following the QbD approach. The addition of PG endowed the HG with the ability to facilitate anticancer activity and with significant potential to be co-encapsulated with other anti-glioblastoma drugs. Our optimized platform provided in vitro proof of the potential of combining the advantages of lipid-based NPs with HG as a promising intranasal delivery system.

## Figures and Tables

**Table 1:** Effect of independent variables (temperature, surfactant, cholesterol) on Z-average, PDI and Zeta potential of 15 runs on design of expert. * Data are presented as average ± SD (*n* = 3 independent measurements).

Number of Runs	Temperature (°C)	Amount of Surfactant (mg)	Amount of Cholesterol (mg)	Z-Average (nm)	PDI	Zeta Potential (mV)
1	45	25	40	150 ± 10	0.30 ± 0.01	−30 ± 8.4
2	20	25	40	220 ± 5.5	0.22 ± 0.02	−29 ± 6.5
3	45	25	40	140 ± 4.5	0.23 ± 0.02	−31 ± 8.4
4	80	10	40	155 ± 5.5	0.25 ± 0.05	−29 ± 8.4
5	45	40	40	500 ± 6.6	0.44 ± 0.07	−5 ± 7.5
6	70	40	20	400 ± 7.8	0.55 ± 0.01	−6 ± 8.5
7 *	70	10	60	120 ± 8.8	0.12 ± 0.08	−38 ± 10.2
8	45	25	40	155 ± 22	0.26 ± 0.09	−29 ± 12
9	45	10	40	200 ± 2.3	0.21 ± 0.08	−29 ± 5.5
10	20	10	20	230 ± 2.4	0.22 ± 0.06	−19 ± 6.5
11	45	25	40	160 ± 40	0.25 ± 0.08	−28 ± 10
12	45	25	40	145 ± 20	0.18 ± 0.05	−28 ± 10.2
13	20	40	60	600 ± 12	0.46 ± 0.01	−4 ± 3.3
14	45	25	20	222 ± 10	0.23 ± 0.02	−20 ± 5.5
15	45	25	60	190 ± 14	0.22 ± 0.02	−19 ± 6.2

* Parameters of optimized formulation.

**Table 2:** Main physicochemical characteristics of hydrogels at various hyaluronic acid concentrations.

HA Content (% *w*/*v*)	pH Value	Drug Contents (%)	Spreadability (mm^2^)	Mucoadhesion Displacement (mm) after 7 h	Viscosity Cross-Linked (Pas)	Viscosity Non-Cross-Linked (Pas)
0.5	5.3 ± 0.2	78 ± 2.5	222.45 ± 0.22	20 *	0.112	0.181
1	5.2 ± 0.3	82 ± 3.3	360 ± 0.33	20	1.88	2.11
2	5.5 ± 0.4	80 ± 1.4	320 ± 0.44	10	14.29	15.45
3	5.9 ± 0.6	79 ± 4.2	340 ± 0.012	1	66.34	157

* After 2 h maximum displacement on agar-mucin plate was already reached.

## Data Availability

Data is contained within the article and supplementary material.

## Supplementary Materials

The following are available online at https://www.mdpi.com/article/10.3390/ph14070696/s1, Figure S1: Interdependence rating amongst QTPP—CQA (a) and CPP/CMA—CQA (b) elements, Figure S2: 3D surface plot (a) and one-factor interaction (b) graph showing the effects of surfactant and cholesterol on Particle Size, PDI and Zeta potential, Table S1: Kinetic parameters of in vitro drug release.

## Abbreviations

ANOVA	one-way analysis of variance
BBB	blood–brain barrier
CNS	central nervous system
CPPs	critical process parameters
CMA	critical materials attributes
CQA	critical quality attributes
CCD	central composite design
°C	centigrade
EE	encapsulation efficiency
FTIR	Fourier-transformed infrared spectroscopy
GA	glutaraldehyde
HA	hyaluronic acid
HG	hydrogel
HA-HG	hyaluronic acid—hydrogel
HPLC	high proficiency liquid chromatography
H_2_O_2_	hydrogen peroxide
ICH	International Conference on Harmonization
KBr	potassium bromide
kDa	kilo Dalton
LC	loading capacity
LOQ	limit of quantification
LOD	limit of detection
mV	millivolt
mm	millimol
mA	milliampere
mPa	millipascal
mg	milligram
mm^2^	square millimeter
Mw	molecular weight
nm	nanometer
ppm	parts per million
PBS	phosphate buffer saline
PG	propyl gallate
PG-SLNs	PG-solid lipid nanoparticles
PDI	polydispersity index
Pas	pascal
QbD	Quality by Design
QTPP	quality target product profile
RA	risk assessment
SLNs	solid lipid nanoparticles
SEM	scanning electron microscopy
SLNs-HGnCL	SLNs non-cross-linked HG
SLNs-HGCL	SLNs cross-linked HG
TC-P	Transcutol-P
µm	micrometer
w/v	weight/volume
XRPD	X-ray powder diffractograms
