# Supplementary material for: Development and Characterization of n-Propyl Gallate Encapsulated Solid Lipid Nanoparticles-Loaded Hydrogel for Intranasal Delivery
Source: Pharmaceuticals (Basel). 2021 Jul 19;14(7):696. doi: 10.3390/ph14070696 (PMC8308668; doi:10.3390/ph14070696)
Supplement: Supplementary file 1 [file pharmaceuticals-14-00696-s001.zip › pharmaceuticals-1263644-supplementary.pdf]

Development and Characterization of *n*-Propyl gallate Encapsulated Solid Lipid Nanoparticles-Loaded Hydrogel for Intranasal Delivery

Fakhara Sabir [1], Gábor Katona [1], Ruba Ismail [1,2], Rita Ambrus [1], Bence Sipos [1], Ildikó Csóka [1]*

[1] Institute of Pharmaceutical Technology and Regulatory Affairs, Faculty of Pharmacy, University of Szeged, Eötvös Str. 6, H-6720 Szeged, Hungary; fakhra.sabir@gmail.com (F.S.); katona.gabor@szte.hu (G.K.); ruba.ismail@szte.hu (R.I.); ambrus.rita@szte.hu (R.A.); sipos.bence@szte.hu (B.S.) csoka.ildiko@szte.hu (I.C.)
[2] Faculty of Science and Informatics, Department of Applied & Environmental Chemistry, Rerrich Béla sqr. 1, H-6720, Szeged, Hungary
*Correspondence: csoka.ildiko@szte.hu, Tel. +36-62-546-116

Supplementary material

(a)

CQA \ QTPP	Route of administration	Indication	Dissolution profile	Permeability profile	Stability	Brain distribution
Z-average, PDI	M	H	H	H	M	M
Zeta potential	M	L	H	H	H	M
Encapsulation efficiency	M	M	H	M	L	M
Mucoadhesion	H	L	L	L	L	L
Viscosity	H	L	L	L	L	L
Swelling properties	M	L	M	M	L	L

(b)

	Material attributives				Subprocesses			
CQA \ CPP/CMA	Ratio of organic phase	Cholesterol content	Tween 80 content	Temp. at dissolution	Sonication	Injection and stirring	Evaporation of organic solvents	Freeze-drying
Z-average, PDI	M	H	H	H	M	M	M	M
Zeta potential	M	H	H	H	M	M	M	M
Encapsulation efficiency	M	H	H	M	M	L	L	L
Mucoadhesion	L	M	M	L	L	L	L	L
Viscosity	L	L	M	M	L	L	L	L
Swelling properties	L	M	M	L	L	L	L	L

Figure S1. Interdependence rating amongst QTPP – CQA (**a**) and CPP/CMA – CQA (**b**) elements. The relations are presented on a 3-grade scale as: "H" as high, marked with red; "M" as medium, marked with orange; and "L" as low, marked with green.

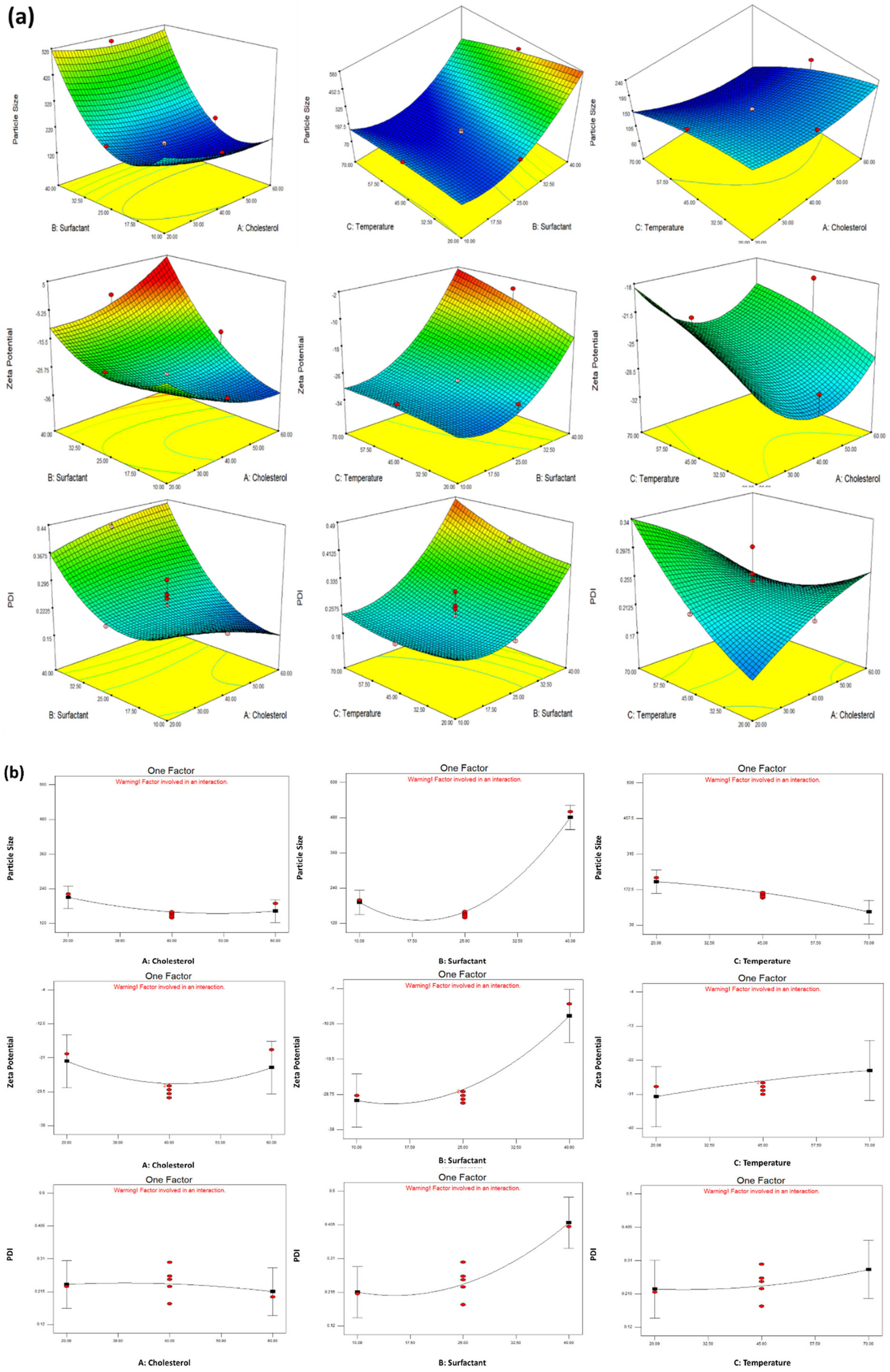
(a)
Particle Size
Zeta Potential
PDI
B: Surfactant
A: Cholesterol
C: Temperature
(b)
One Factor
Warning! Factor involved in an interaction.
A: Cholesterol
B: Surfactant
C: Temperature

Figure S2. 3D surface plot (a) and one-factor interaction (b) graph showing the effects of surfactant and cholesterol on Particle Size, PDI and Zeta potential

Table S1. Kinetic parameters of in vitro drug release

Kinetic Model	Kinetic Parameters	PG dispersion	PG-SLNs	1% *w/v* SLNs-HGnCL
Zero order	k_0 (μg min^{-1})	0.391	0.094	0.263
	R^2	0.791	0.8158	0.8803
	$t_{0.5}$ (min)	1950.83	467.64	1315.63
First order	$k_1 \times 10^{-3}$ (min^{-1})	0.191	0.419	1.178
	R^2	0.8463	0.9299	0.9318
	$t_{0.5}$ (min)	3638.37	1655.35	588.311
Higuchi model	k_H (μg $min^{-1/2}$)	12.007	32.747	21.461
	R^2	0.9417	0.96	0.9783
	$t_{0.5}$ (min)	6764.02	909.35	2117.26
Korshmeyer-Peppas model	k_{K-P} (min^{-n})	1.045	8.876	6.772
	n	0.43	0.31	0.28
	R^2	0.9567	0.8462	0.8552
	$t_{0.5}$ (min)	2065.96	812.34	1154.59
Hixon-Crowell model	k_{H-C} ($μg^{1/3}$ min^{-1})	0.011	0.163	0.039
	R^2	0.9838	0.8535	0.9391
	$t_{0.5}$ (min)	12090.3	1979.38	2688.46
Best fit		Hixon-Crowell model	Higuchi model	Higuchi model
